# Review of microbiota gut brain axis and innate immunity in inflammatory and infective diseases

**DOI:** 10.3389/fcimb.2023.1282431

**Published:** 2023-10-04

**Authors:** Chongshan Yuan, Yuhong He, Kunyu Xie, Lianjun Feng, Shouyang Gao, Lifu Cai

**Affiliations:** ^1^ Department of Obstetrics, China-Japan Union Hospital of Jilin University, Changchun, Jilin, China; ^2^ Department of Clinical Veterinary Medicine, College of Veterinary Medicine, Jilin University, Changchun, Jilin, China

**Keywords:** microbiota gut brain axis, inflammatory diseases, infective diseases, pathogenesis, innate immune

## Abstract

The microbiota gut brain (MGB) axis has been shown to play a significant role in the regulation of inflammatory and infective diseases. Exploring the structure and communication mode of MGB axis is crucial for understanding its role in diseases, and studying the signaling pathways and regulatory methods of MGB axis regulation in diseases is also of profound significance for future clinical research. This article reviews the composition, communication mechanism of MGB axis and its role in inflammatory and infective diseases, including Parkinson’s disease (PD), Alzheimer’s disease (AD), multiple sclerosis (MS), autism spectrum disorder (ASD), depression, psoriasis, irritable bowel syndrome (IBS), and inflammatory bowel diseases (IBD). In addition, our investigation delved into the regulatory functions of the inflammasome, IFN-I, NF-κB, and PARK7/DJ-1 innate immune signaling pathway in the context of inflammatory and infective diseases. Ultimately, we discussed the efficacy of various interventions, including fecal microbiota transplantation (FMT), antibiotics, probiotics, prebiotics, synbiotics, and postbiotics, in the management of inflammatory and infective diseases. Understanding the role and mechanism of the MGB axis might make positive effects in the treatment of inflammatory and infective diseases.

## 1 Introduction

With widespread study on germ-free (GF) animal models, the connection between gut microbiota and innate immunity has also been widely recognized (Campbell et al., 2023). Gut microbiota may aggravate the progress of inflammatory and infective diseases by destroying the innate immune system (Wastyk et al., 2021). Exploring the interaction between gut microbiota and innate immune system may help to reveal the causes of inflammatory and infective diseases. From the perspective of central nervous system (CNS) perception of hunger and short-term regulation of food intake by the intestine, the existence of bidirectional gut brain communication seems obvious (Morais et al., 2021). The specific linkage between the gut and the CNS is known as the “gut brain axis” and is composed of bidirectional exchanges between the two, which has been a study focus for decades (Dinan and Cryan, 2017). The development of sequencing technologies such as 16S ribosomal RNA and metagenomics promoted in-depth exploration of gut microbiota (Lagier et al., 2018). According to reports, the diversity of gut microbiota is related to the gut and innate immune/inflammatory responses, which may further regulate neuroinflammation and neurodegeneration in the CNS (Pellegrini et al., 2020). Considering the crucial role of gut microbiota in maintaining organ and system homeostasis, the concept of “microbiota gut brain (MGB) axis” has also emerged. More and more evidence emphasizes the role of the MGB axis in regulating brain and intestinal function, and its correlation with inflammatory and infective diseases has also been received attention (Asadi et al., 2022). Despite increasing evidence, there is still a significant knowledge gap in the exact mechanisms by which MGB axis regulates inflammatory and infective diseases. This review provided an overview of the structure and communication mechanisms of MGB axis. Additionally, we explored the role and regulatory mechanisms of MGB axis in inflammatory and infective diseases. Finally, we summarized methods for regulating inflammatory and infective diseases. The purpose of this review is to provide new insights for MGB axis and innate immune system to regulate inflammatory and infective diseases.

## 2 The microbiota gut brain axis

**Figure 1:**
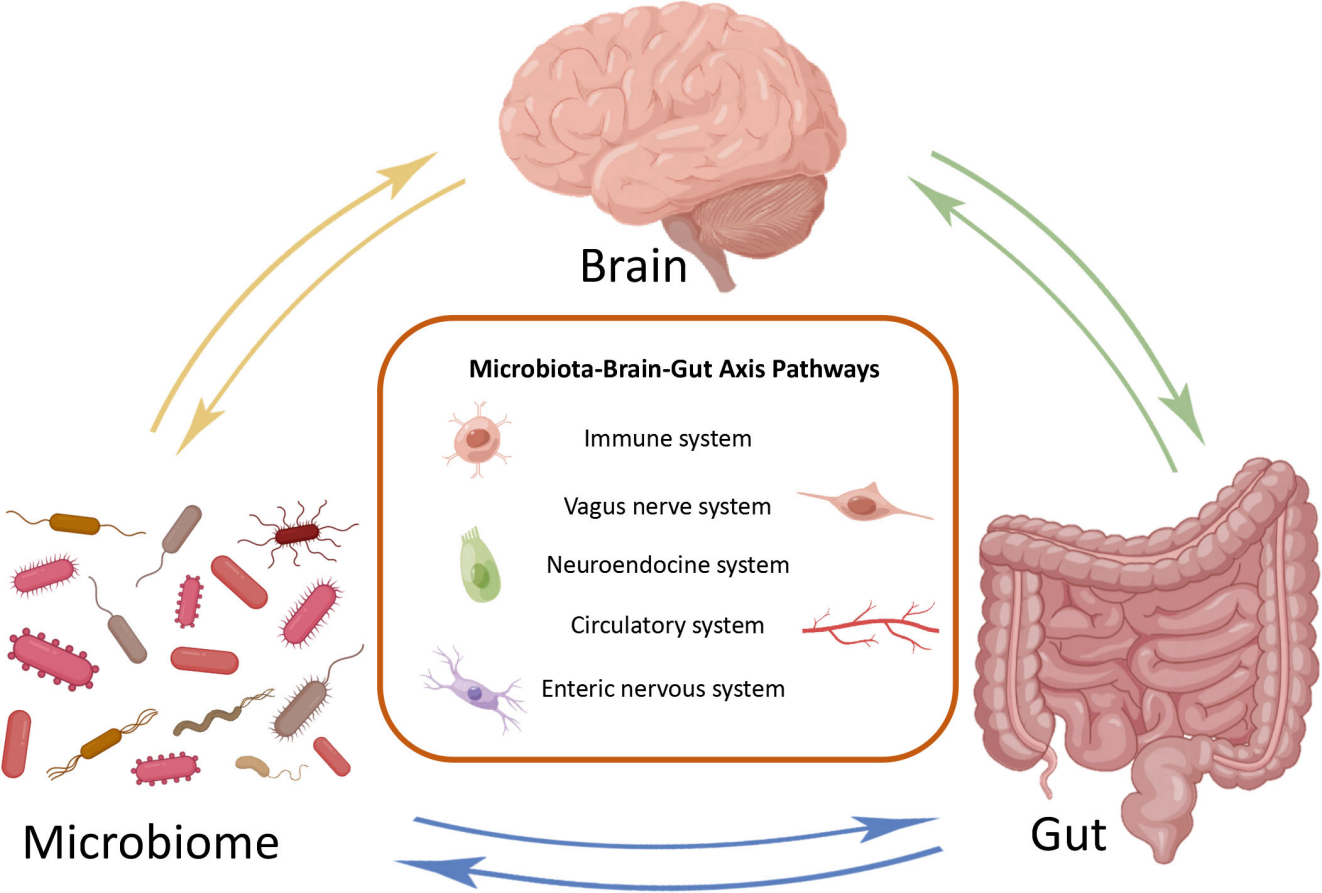
Bidirectional communication of microbial gut brain axis. The brain, gut, and microbiome constitute three nodes in bidirectional communication. Immune system, vagus nerve system, neuroendocrine system, circulatory system and enteric nervous system are the main ways of bidirectional communication.

The MGB axis is composed of gut microbiota, CNS, enteric nervous system (ENS), parasympathetic nerves, sympathetic nervous system, neuropeptides, and immune barrier (Tan, 2023). It is defined as a bidirectional communication between the brain and gut bacterial communities formed by multiple systems, playing an significant role in maintaining normal system functionality (**Figure 1**) (Cryan et al., 2019). The potential mechanism of the MGB axis mainly relies on gut microbiota, especially the interactions between immune-bacterial with intestinal epithelial barrier (IEB), innate immune system, and neural pathways (Pellegrini et al., 2018). In this process, inflammasomes play a role as immune outposts in sensing intestinal bacteria and regulating brain physiology (Rutsch et al., 2020). Therefore, understanding the composition of the gut brain axis, including gut microbiota, nervous system and immune barrier, is particularly important for exploring its regulation of inflammatory and infective diseases.

### 2.1 Gut microbiota

**Figure 2:**
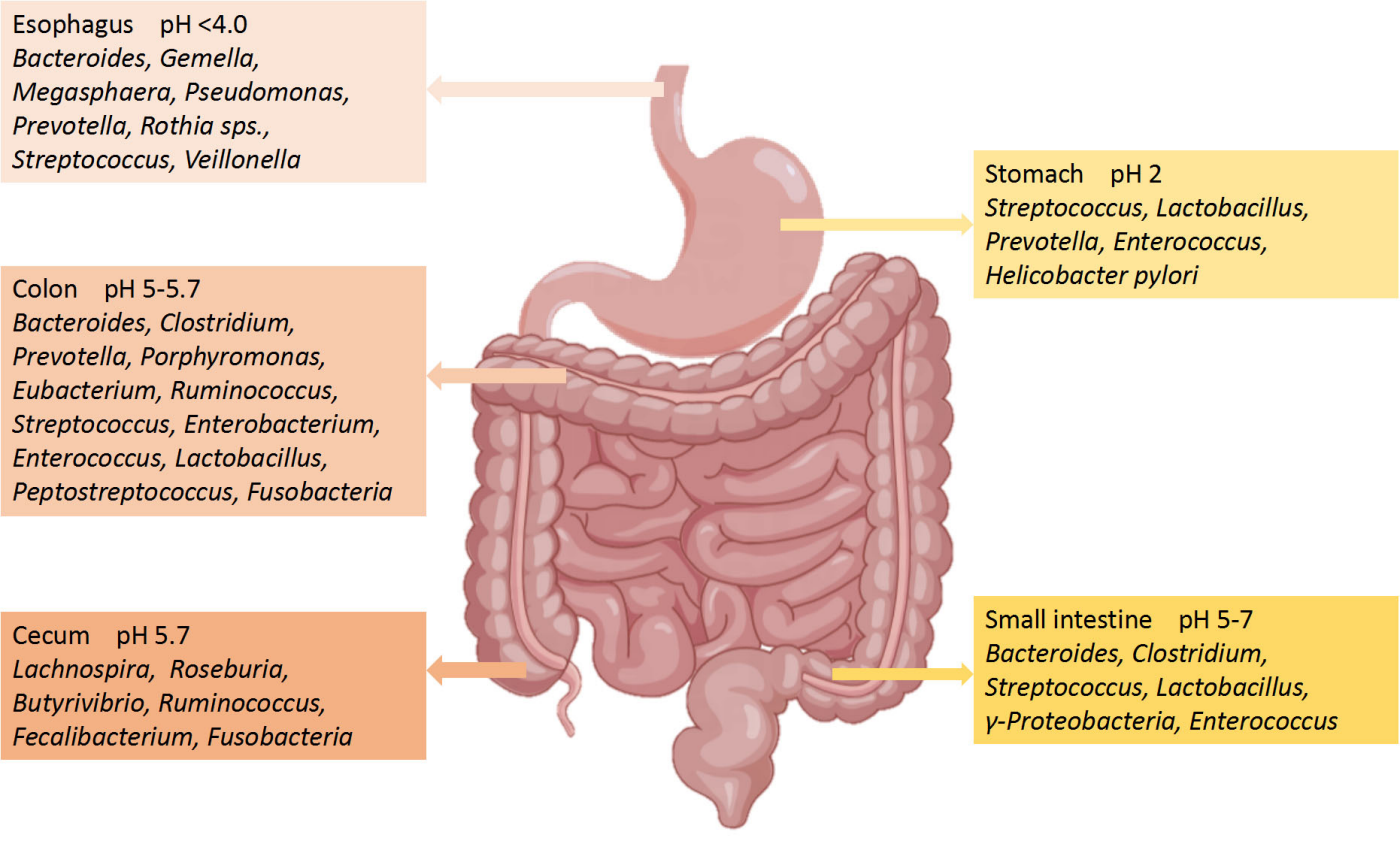
Main microorganisms and pH in different regions of the gastrointestinal tract.

The number of microorganisms in the body is trillions, 1.3 times that of human cells. Adult microorganisms can reach about 1 kg, with most of them exist in the intestines, collectively known as the gut microbiota (Krautkramer et al., 2021). The gut microbiome is composed of commensal bacteria, fungi, phages, yeasts, parasite archea, and virus (Nicholson et al., 2019), with a species count of over 1000. The microbial biomass in the cecum and proximal colon is the highest, while the microbiota in the small intestine is basically consistent to that in the large intestine (Tierney et al., 2019). The gut microbiota has a positive role in regulating innate immunity and inflammation (Campbell et al., 2023). Although the human gut microbiota is easily affected by many factors, the main bacterial species include two main phyla: *Firmicutes* (51%) and *Bacteroidetes* (48%) (Demirci et al., 2020). The remaining 1% consists of *Proteobacteria*, *Actinobacteria Fusobacteria* and other phyla (Khan et al., 2020). *Bacteroidetes* include *Bacteroides* and *Prevotella*, while *Clostridium*, *Faecalibacterium* and *Ruminococcus* represent *Firmicutes* (Chen T. et al., 2021). The composition of the main microorganisms in different regions of the gastrointestinal tract is shown in **Figure 2** (Jandhyala et al., 2015). Even though the composition of gut microbiota has been a research focus in recent years, the characteristics and functions of microbiota still need further study (Balmus et al., 2020). Gut microbiota can regulate the development of inflammatory bowel disease (IBD), innate immune diseases and neuroinflammatory diseases through immune signals (Gilbert et al., 2018). Further studies have confirmed that the development of nerves and the activation of microglia have been shown to depend on the gut microbiota (Li et al., 2023). It can be seen that the homeostasis of gut microbiota has a positive effect on regulating the immune and nervous systems.

### 2.2 Nervous system

Given the role of gut microbiota in the nervous system, understanding the composition of the nervous system can serve as a reference for regulating inflammatory and infective diseases. CNS and peripheral nervous system are the main components of the nervous system. The brain and spinal cord constitute the CNS (Fu, 2018), while the peripheral nervous system consists of brain nerve, spinal nerve and autonomic nervous system (ANS), of which the ANS includes sympathetic nervous system (SNS), parasympathetic nervous system (PNS) and ENS (Sousa et al., 2017). ANS mainly regulates the physiological stability of the host by controlling visceral activity, gland secretion, and communication between gut and CNS. Although SNS and the PNS antagonize each other, they can synergistically affect peripheral effector organs under the control of the CNS (Foster et al., 2017). As the main part of the PNS, the vagus nerve can sense the relevant signals of the intestinal tract and microbiota and transmit them to the CNS, subsequently inducing adaptive or inappropriate responses (Bonaz et al., 2018). ENS has many likenesses with CNS, hence it is called the “second brain”. Although ENS can independently regulate gastrointestinal peristalsis without being controlled by CNS, it is also regulated by the brain, ANS, immune system, and gut microbiota (Almeida et al., 2022). Although the vagus nerve and ENS are functionally interconnected, the mechanism of their interaction needs further study.

### 2.3 Immune barrier

The blood brain barrier (BBB) and IEB play important roles in maintaining the stability of the MGB axis including gut microbiota and nervous system, and resisting the invasion of inflammatory factors. The integrity of BBB is related to the stability of the brain environment, while the integrity of the IEB is related to the homeostasis of gut microbiota (Langen et al., 2019). When the barrier is disrupted, it can lead to an increase in its permeability, which can lead to inflammatory reactions and changes in the gut microbiota (Suzuki, 2020). In the case of increased intestinal inflammatory response, harmful bacteria, toxic metabolites, and small molecule substances can be directly released into the bloodstream through IEB (Obrenovich, 2018). During this process, lipopolysaccharides (LPS) produced by microbiota is translocated throughout the body, and the consequent pro-inflammatory cytokines activate the systemic immune system, ultimately increasing the permeability of the BBB and damaging brain tissue (Logsdon et al., 2018). On the other hand, the destruction of the BBB can further promote systemic chronic inflammation caused by the gut microbiota (Sweeney et al., 2018). Therefore, the integrity of IEB and BBB is important in preventing inflammatory and infective diseases caused by gut microbiota.

## 3 Signaling mechanisms of microbiota gut brain axis communication

The MGB axis includes gut microbiota, nervous system and immune barrier, but exploring the communication mechanisms within it is of positive significance for in-depth research on its regulation of inflammatory and infective diseases. From the perspective of the CNS being able to perceive hunger and regulate food intake, the existence of bidirectional communication between the gut and brain seems to be easily detected (Socala et al., 2021). Study shows that microbiome has become an indispensable participant in enterocerebral communication, and gut microbiome is important in maintaining the integrity of BBB, the development of CNS, neurogenesis, neurotransmission, and immune cell activity (Lin et al., 2023). Neurons, metabolites, and innate immune signaling mediators are the connections between the gut microbiota and CNS. When the microbiota is disrupted, it leads to changes in BBB permeability and neuroinflammation (Rutsch et al., 2020).On the contrary, the CNS can alter the composition of the gut microbiota through the expression of virulence genes induced by external factors (Morais et al., 2021). At the same time, ANS and ENS indirectly affect the gut microbiome by controlling movement, immune regulation, and endocrine function (Osadchiy et al., 2019). Many microbiota can directly secrete neurotransmitters and act on the CNS by stimulating epithelial cells. When the production of neurotransmitters is disordered, it will promote the development of inflammation and infectious diseases (Long-Smith et al., 2020). However, the examination of the MGB axis is mostly limited to cross-sectional studies, and there is a significant gap in the basic mechanism of bidirectional communication between the MGB axis. Further research is needed on pathways such as vagus nerve activation, immune system, metabolites, and neurotransmitters.

### 3.1 Vagus nerve

**Figure 3:**
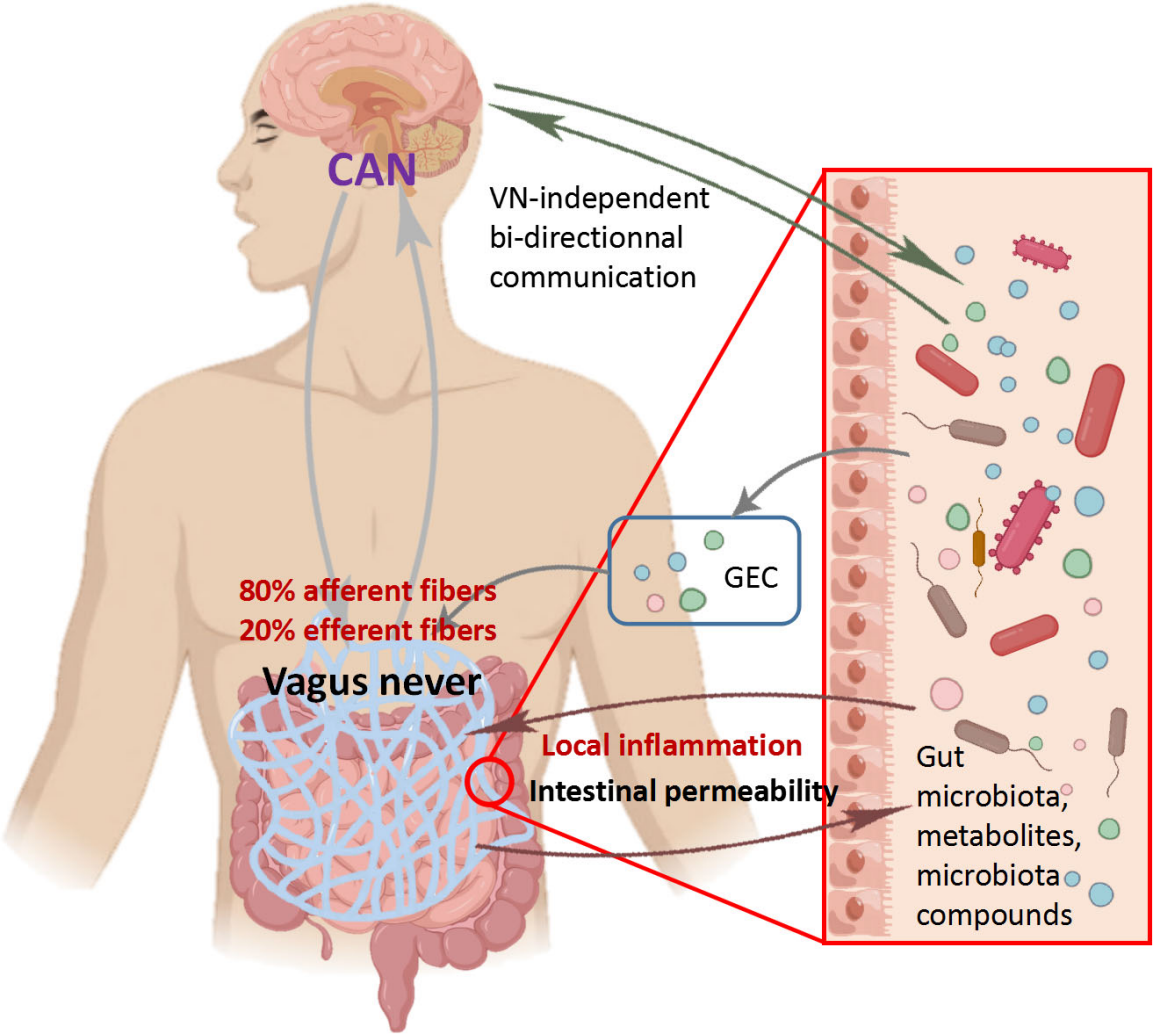
Role of vagus nerve in communication between central nervous system and microbiota Vagus nerve afferent fibers can sense the stimulation of microbial components through gut endocrine cells (GEC). The Central Autonomous Neural Network (CAN) can transmit signals from vagus nerve afferent fibers to the CNS. The inflammatory response further stimulates efferent fibers. On the contrary, vagus nerve efferent fibers reduce digestive inflammation, reduce intestinal permeability, and indirectly regulate the composition of gut microbiota through tight junction reinforcement.

The vagus nerve, which consists of 80% afferent fibers and 20% efferent fibers, is the main component of the PNS. The vagus nerve is one of the key communication modes between the gut and brain, heavily related to the MGB axis signal (Fulling et al., 2019). The afferent fibers of the vagus nerve are widely present in the intestine, but do not interact directly with the gut microbiota (Bonaz et al., 2018). However, the vagus nerve can sense microbial signals through bacterial metabolites and transmit the information to the CNS for response (**Figure 3**) (Bonaz et al., 2018). The glutamate and serotonin released by intestinal endocrine cells can activate the vagus nerve and provide feedback to the CNS (Kaelberer et al., 2018). It is worth noting that compared to many possible pathways, the vagus nerve, as the most direct pathway for bidirectional communication on the MGB axis, has become a research focus on inflammatory diseases. Before the widespread use of drugs to treat *Helicobacter pylori* disease, vagotomy was commonly used for surgical treatment of peptic ulcers disease (Wu et al., 2020). Inhibiting the vagus nerve can disrupt the homeostasis of the gut microbiota, thereby promoting inflammatory diseases such as irritable bowel syndrome (IBS) and IBD (Oswiecimska et al., 2017). In preclinical studies, many beneficial effects of *Lactobacillus rhamnosus* disappeared in mice undergoing vagotomy (Liu et al., 2021). Recent studies have fully demonstrated that the vagus nerve regulates emotions and behavior by influencing CNS responses (Fulling et al., 2019). The disconnection of vagus nerve can block the central signaling in species belonging to the *Lactobacillus* and *Bifidobacterium* genera, resulting in impaired emotional regulation (Zhao et al., 2023). In summary, the vagus nerve plays an indispensable role in the information transmission of gut microbiota and CNS. It is speculated that the integrity of the vagus nerve is also important for the bidirectional communication of MGB axis.

### 3.2 Immune mechanism

Except vagus nerve, the immune system also plays a crucial role in promoting bidirectional communication of MGB axis (Muller et al., 2020). In addition, the integrity of the immune system is crucial for maintaining dynamic immunity and protecting the body from pathogenic microorganisms (Agusti et al., 2018). 70% -80% of immune cells in the body exist in mesentery lymph nodes, and its main components include dendritic cells, macrophages, neutrophils, natural killer cells and mast cells (Zhou et al., 2021). Among them, macrophages are widely present in the entire intestine, and the participation of gut microbiota makes macrophages play an important role in regulating bidirectional communication between the gut and neurons (Jarret et al., 2020). When microglia are activated, they can secrete a variety of antigen markers to control neurotransmitters and cause neuroinflammatory reaction (Erny et al., 2015). It is speculated that gut microbiota can regulate innate immunity, adaptive immunity, and inflammatory responses, and may affect the activation of gut-extrinsic sympathetic nerves in the gut through the gut brain circuit. Meanwhile, the homeostasis of gut microbiota plays a positive role in the maturation, health and normal function of microglia (Mossad and Blank, 2021). In addition, gut microbiota seems to be one of the most important factors for microglia maturation and astrocyte activation (Sun et al., 2018). Study has found that GF mice exhibit immune abnormalities such as T cell, B cell populations, and cytokine reduction (Fujita et al., 2020). On the other hand, recolonization of *Bacteroides fragilis* can maintain immune maturation of gut-associated lymphoid tissue (Gomaa, 2020). Pattern recognition receptors (PRRs) such as transmembrane surface or endosome toll-like receptors (TLRs), peptidoglycans (PGN), and cytosolic nucleotidebinding oligomerization domain-like receptors (NLRs) are the key to most innate immune responses, which can mediate the immune response to microorganisms (Yuan M. et al., 2021). As a sensor for the presence of gut microbiota, TLRs can transmit information to ENS, causing changes in the development and function of the gut nervous system. Lack of TLR2 signaling leads to abnormal neurochemical coding in mice ENS, which is reversed after supplementation with TLR2 agonists (Yarandi et al., 2020). The decrease in the expression of several receptors for detecting PGN in the striatum of mice treated with GF and antibiotics. Furthermore, knocking down PGN sensitive receptors can leads to an increase in social ability and behavioral changes in mice (Arentsen et al., 2017). It can be seen that the immune system is closely related to the homeostasis of gut microbiota. In addition, the immune system may be the key to bidirectional communication between the gut microbiota and the nervous system, providing another channel for exploring the communication of the MGB axis.

### 3.3 Metabolites and neurotransmitters

Metabolites and neurotransmitters play an irreplaceable role in regulating bidirectional communication of MGB axis. However, most neurotransmitters produced by microorganisms have a short half-life and limited ability to cross the BBB (Taj and Jamil, 2018). Therefore, it remains to be explored whether these neurotransmitters can reach specific targets and whether they have sufficient concentrations to regulate the CNS (Channer et al., 2023). On the other hand, short chain fatty acids (SCFAs) or neurotransmitters produced by specific bacteria can activate the innate immune system, affecting the CNS and regulating brain physiology by regulating immune/inflammatory cell activity (Foster et al., 2017). Serotonin, SCFAs, and other tryptophan metabolites have been shown to have a significant impact on the stability of ENS and CNS (Brummelte et al., 2017). These metabolites and products can transmit information to the CNS through the nervous system, circulatory system, and immune system (Sonner et al., 2019). There are reports that the diversity and relative abundance of gut microbiota determine the types and concentrations of microbial metabolites and products, and are associated with a range of inflammatory diseases, including Parkinson’s disease (PD), Alzheimer’s disease (AD), and IBD (Bastiaanssen et al., 2019). In summary, the regulation of metabolites and neurotransmitters on inflammatory diseases is multifaceted. At the same time, the relationship between metabolites, neurotransmitters and gut microbiota is obvious, which provides a good idea for regulating the communication of MGB axis.

#### 3.3.1 Tryptophan

Bacteria convert tryptophan from dietary sources into tryptophan by expressing tryptophan decarboxylase. Recent studies have found that tryptamine is an important signaling substance for host microbial interactions, which can affect gastrointestinal motility through the action of 5-HT4R (Bhattarai et al., 2018). However, it is currently unclear whether tryptamine can reach the CNS and regulate its behavior. Kynurenine pathway is the main pathway of tryptophan metabolism. Study has shown that reduced activation of the peripheral canine kynurenine pathway can improve the utilization of tryptophan in GF mice (Wei et al., 2021). When Tryptophan is converted into caninurenine, it can lead to neuroinflammation and be harmful to brain health (Kennedy et al., 2017). The gut microbiota can convert tryptophan into indole, thereby promoting intestinal homeostasis (Gheorghe et al., 2019). It can be seen that exploring the mechanism by which tryptophan produced by gut microbiota regulates the nervous system may provide a new direction for reducing inflammatory response.

#### 3.3.2 Short chain fatty acids

SCFAs are associated with various inflammatory diseases and can affect the integrity of intestinal epithelium, glycolipid metabolism, and immune system homeostasis. The main metabolic product of the gut microbiota is SCFAs, with approximately 95% being acetate, propionate and butyrate (Zaky et al., 2021). The relative abundance of bacteria such as *Clostridia*, *Bifdobacteria*, *Bacteroides*, and *Lactobacillus* is related to the content of SCFAs in the intestine (Xiao et al., 2022). SCFAs can easily enter the circulatory system from the intestine and directly affect the CNS by passing through the BBB *via* monocarboxylate transporters (Wenzel et al., 2020). In addition, SCFAs interacts with enzymes involved in its biosynthesis to affect the production of neurotransmitters in the brain (Zhong et al., 2023). It is reported that the treatment with acetate, propionate and butyrate can restore the morphological defect of microglia in GF mice (Erny et al., 2015). However, other studies have reported that the half-life of SCFAs is only 25 minutes to 3 hours, it is unclear whether these metabolites can regulate neurotransmission in the body (Mandaliya and Seshadri, 2019). Therefore, it is necessary to determine the optimal concentration for it to reach the brain. Looking forward, the intervention and treatment of inflammatory diseases by SCFAs may be a particularly promising candidate.

#### 3.3.3 Histamine

It is reported that most bacteria have the histidine decarboxylase gene, which exists in most bacteria, such as *Lactobacillus*, *Streptococcus* and *Enterococcus*, and is the key gene for histamine production (De Palma et al., 2022). Histamine induces anti-inflammatory responses through the action of H4R receptors, which are particularly crucial in the CNS. However, depending on the receptors it acts on, histamine can also have pro-inflammatory properties by promoting the production of various chemokines (Kim et al., 2022). It was reported that histamine may be associated with neuroinflammatory diseases, which may exacerbate the severity of AD (Flores-Clemente et al., 2021). The concentration and localization of histamine receptors in the central or systemic regions have a broad impact on the occurrence of neuroinflammation, and regulating the gut microbiota to cause changes in histamine has become a possible route for treating inflammation (Carthy and Ellender, 2021).

#### 3.3.4 Serotonin

As a crucial transmitter of neurons, Serotonin (5-HT) can affect the development and function of both the ENS and CNS through endocrine and paracrine signaling processes (Xiao et al., 2021). It has been observed that 5-HT can promote the secretion of cytokines by lymphocytes and monocytes, and send information to the CNS by stimulating the vagus nerve (McVey et al., 2019). It is worth mentioning that the microbiota plays an important regulatory role in the production of 5-HT. Based on metabolomics data mining, it was found that approximately 20% of microorganisms have the ability to synthesize 5-HT (Valles-Colomer et al., 2019). On the contrary, enterochromaffin cells (ECCs) contain over 90% of human 5-HT, and ANS can release 5-HT into the intestinal by activating ECCs and alter the characteristics and functions of the gut microbiota (Sgritta et al., 2019). It is reported that compared to normal mice, the 5-HT levels of GF mice are significantly reduced (Xiao et al., 2021). It has been shown that specific spore forming bacteria increase the levels of 5-HT in the colon and serum of GF mice. The increase in SCFAs concentration increased the production of 5-HT and improved GF related intestinal dysfunction by upregulating the expression of Tph1 in ECCs (Beyder, 2018). According to reports, IBS can increase the content of 5-HT in the blood, which further leads to the degree of visceral pain in patients (Luo et al., 2021). Similarly, 5-HT released by endothelial cells may lead to the occurrence of IBS abdominal pain by regulating vagus and inflammatory responses (Gao et al., 2022). Therefore, elucidating the relationship between 5-HT and gut microbiota has a promoting effect on the treatment of inflammatory diseases.

#### 3.3.5 Ghrelin

When the stomach is emptied, the secretion of ghrelin in the gastrointestinal tract increases, and gut microbiota dynamics can regulate ghrelin secretion. When taking *Bifidobacterium*, the secretion of ghrelin also decreases (Chen C. et al., 2022). In addition, as a neuropeptide in the CNS, gastrin is an crucial regulator of nerve function and inflammation (Yuan MJ. et al., 2021). The protective effect of ghrelin on neuroinflammatory diseases such as AD and PD has also been extensively demonstrated. AD can reduce the secretion of ghrelin in the brain, and the increase of ghrelin can promote the synaptic plasticity of AD patients, save memory defects, and inhibit excessive inflammatory response (Russo et al., 2022). In PD, ghrelin provides protection against the toxic model of PD by protecting dopaminergic cells and mitochondrial function (Wang et al., 2021). Obviously, ghrelin is crucial for regulating CNS and is closely related to the gut microbiota. It is speculated that ghrelin may regulate neuroinflammatory diseases through the MGB axis.

## 4 Microbial brain gut axis regulates inflammatory and infective diseases

When inflammatory or infective diseases occur, the immune system, nervous system, gut microbiota, and metabolites are all involved in this process. The homeostasis of gut microbiota plays an important role in regulating inflammatory and infective diseases. When the relative microbial population is imbalanced, unhealthy signals can be sent to the brain and cause mild systemic inflammation or infectious diseases (Noble et al., 2017). Numerous studies have found that gut microbiota is associated with inflammatory and infective diseases including PD, AD, IBD, etc (Haneishi et al., 2023). The microbial composition of patients with inflammatory and infective diseases is shown in **Table 1**. Directly targeting the gut microbiota is a good breakthrough for regulating the MGB axis. and the bidirectional communication of the MGB axis provides research directions for regulating neurological and intestinal inflammatory diseases (Agirman and Hsiao, 2021). Although research on the MGB axis is still in its early stages, it provides a potentially crucial approach for intervening in inflammatory and infective diseases.

**Table 1:** The effect of inflammatory and infective diseases on gut microbiota.

Diseases	Study design	Key Findings	Ref.
Parkinson’s Disease (PD)	PD: 223 Controls: 137	*Akkermansia*, *Catabacter*, and *Akkermansiaceae*, increased while *Roseburia*, *Faecalibacterium*, and *Lachnospiraceae* decreased in PD.	(Nishiwaki et al., 2020)
Parkinson’s Disease (PD)	15 case-control studies	*Bifidobacteriaceae*, *Ruminococcaceae*, *Verrucomicrobiaceae*, and *Christensenellaceae* increased while *Prevotellaceae*, *Faecalibacterium*, and *Lachnospiraceae* decreased in PD.	(Shen et al., 2021)
Parkinson’s Disease (PD)	Meta-analysis 10 currently available 16S microbiome datasets	*Lactobacillus*, *Akkermansia*, and *Bifidobacterium increased while Lachnospiraceae* and *Faecalibacterium* decreased in PD.	(Romano et al., 2021)
Parkinson’s Disease (PD)	Meta-analysis PD: 5043 controls: 23,449	*Helicobacter pylori* increased in PD.	(Dardiotis et al., 2018)
Alzheimer’s disease (AD)	AD: 427 controls: 378	*Proteobacteria*, *Bifidobacterium* and *Phascolarctobacterium* increased while *Firmicutes*, *Clostridiaceae*, *Lachnospiraceae* and *Rikenellaceae* decreased in AD.	(Hung et al., 2022)
Multiple sclerosis (MS)	MS: 71	*Akkermansia muciniphila* and *Acinetobacter calcoaceticus* increased in MS.	(Cekanaviciute et al., 2017)
Autism spectrum disorder (ASD)	18 participants	*Bifidobacteria* and *Prevotella* increased in ASD.	(Kang et al., 2019)
Depression	carbohydrate diet treatment: 22 low-fat diet treatment: 23	*Roseburia*, *Ruminococcus*, and *Eubacterium* dcreased in depression.	(Ren et al., 2020)
Depression	Depression: 36 Controls: 37	*Actinobacteria*, *Firmicutes*, *Bifidobacterium* and *Blautia* increased while *Prevotella* dcreased in depression.	(Chung et al., 2019)
Irritable bowel syndrome (IBS)	24 studies from 22 articles	*Enterobacteriaceae*, *Lactobacillaceae*, and *Bacteroides* increased while *Clostridiales*, *Faecalibacterium*, and *Bifidobacterium* decreased in IBS.	(Pittayanon et al., 2019)
Irritable bowel syndrome (IBS)	IBS-D: 120 Controls: 63	*Prevotella*, *Clostridiales*, and Roseburia, increased while *Veillonellaceae*, *Bacteroides coprocola*, and *Bifidobacteriales* dcreased in IBS-D.	(Yao et al., 2023)
Irritable bowel syndrome (IBS)	1,340 participants were included for analysis	*Escherichia coli* and *Enterobacter* increased while *Lactobacillus* and *Bifidobacterium* dcreased in IBS.	(Wang L. et al., 2020)
Irritable bowel syndrome (IBS)	Review of 13 articles IBS: 360 Controls: 268	*Lactobacillus*, *Bifidobacterium*, and *Faecalibacterium prausnitzii* dcreased in IBS.	(Liu HN. et al., 2017)
Inflammatory bowel disease (IBD)	CD:31 UC: 22 Controls: 19	*Clostridiales* dcreased in IBD.	(Lee et al., 2017)

### 4.1 Parkinson’s disease

PD is a common neuroinflammatory disease, with tremors, muscle rigidity, motor delay, and abnormal gait as its main clinical manifestations (Tolosa et al., 2021). Complex genetic and environmental factors make clinical treatment less effective. PD can lead to functional damage to dopaminergic neurons in the substantia nigra, as well as deposition of α-synuclein and Lewy bodies (Schneider and Alcalay, 2017). Emerging evidence shows that PD may transmit signals from the gut to the brain through α-synuclein in the ENS, in which the gut or vagus nerve may play an important role (Arotcarena et al., 2020). Chemicals such as rotenone (Perez-Pardo et al., 2018) and paraquat (Anselmi et al., 2018) can induce PD like symptoms in mice by regulating the accumulation of α-synuclein in dorsal motor nucleus of the vagus nerve and substantia nigra, further demonstrates the important role of the gut brain axis in regulating PD. Notably, disordered gut microbiota can lead to increased deposition of synaptic nucleoprotein, leading to inflammation and further PD (Caputi and Giron, 2018). It is interesting that nearly 80% of PD patients experience constipation, so it is speculated that the cause of the disease begin in the gut microbiota (Zhu et al., 2022). *Prevotellaceae* and *Enterobacteriaceae* have been proven to be biomarkers for diagnosing PD. When PD occurs, the abundance of the former decreases, while the latter significantly increased after the onset of PD. In this case, focusing on changes in the gut microbiota may improve understanding of the occurrence of PD. Remarkably, the study found a 90.3% correlation between the abundance of *Prevobacteriaceae* and constipation with PD. In addition, transplantation of PD patients’ feces into mice can lead to neuroinflammation, further proving the association between gut microbiota and PD (Zhao et al., 2021). Antibiotics, probiotics or truncal vagotomy may be effective strategies for treating PD, although clinical data that can demonstrate the beneficial effects of these methods is still very limited (Liu B. et al., 2017). Moreover, metabolites of gut microbiota, such as SCFAs, amine, folic acid and gastrin, have beneficial effects in treating or regulating the development of PD, which requires further studies (Zheng SY. et al., 2021). In summary, regulating the nervous system and gut microbiota in the MGB axis may be key to the treatment of PD.

### 4.2 Alzheimer’s disease

AD typically occurs in elderly people before the age of 65, with less than 1% of familial AD cases (Murdock and Tsai, 2023). The loss of neurons, damage of synaptic function, and deposition of amyloid-β (Aβ) protein in neurons are the main pathological features of AD (Shi et al., 2022). AD can affect a wide range of areas in the cerebral cortex and hippocampus, showing serious CNS dysfunction in learning, memory and behavioral problems, and seriously affecting daily activities (Kesika et al., 2021). Previous studies have shown that infections with spirochetes, fungi, and Chlamydia pneumoniae can cause CNS inflammation, which in turn can trigger AD (Stojkovic et al., 2020). Despite extensive research on AD, the therapeutic mechanism of AD remains inconclusive (Bachurin et al., 2018). Recent studies have found that the secondary bile acid produced by gut microbiota is believed to be related to AD and mild cognitive impairment patients (Nho et al., 2019). In addition, the gut microbiota can improve cognitive impairment in elderly people by regulating Aβ load (Cattaneo et al., 2017; Manderino et al., 2017). Study has found that there was a significant increase in *Verrucomicrobia* and *Proteobacteria*, *aswellas* in the feces of AD mice, while *Ruminococcus* and *Butyricicoccus* significant decreased, indicating that AD is related to the gut microbiota. The decrease in SCFAs levels further indicates that many metabolic pathway changes are related to AD (Nagarajan et al., 2023). Likewise, it was found that the proportion of *Firmicutes* and *Bacteroidetes* in the intestinal tract of mild AD patients changed, and the richness and diversity of gut microbiota also decreased (Vogt et al., 2017). It has been confirmed that the metabolites of the microbiota are related to the activation of Aβ and NLRP3 inflammasome pathway (Shukla et al., 2021). At the same time, the large deposition of Aβ can catalyze the release of pro-inflammatory molecules throughout the body, causing the progression of AD to worsen (Honarpisheh et al., 2020). Other studies have found that the activation of microglia can induce the pathological process of AD by promoting the deposition of Aβ. When Aβ is deposited, microglia will eventually cause neuroinflammation and worsen AD by releasing various proinflammatory mediators (Wang et al., 2022). In addition, long-term use of broad-spectrum antibiotics can reduce the deposition of Aβ in AD mice and reduce the occurrence of AD (Kumari and Deshmukh, 2021). With the deepening of research, it can be inferred that the MGB axis plays an important role in AD, and targeted regulation of gut microbiota metabolites and microglia may be an effective method to alleviate AD.

### 4.3 Multiple sclerosis

Multiple sclerosis (MS) is a multiple inflammatory demyelinating disease caused by the disorder of immune system and gut microbiome (Jayasinghe et al., 2022). T cells can participate in the process of MS by regulating the nervous system. When the immune activity of T cells is suppressed, it can exacerbate abnormal autoimmune reactions, thereby causing adverse effects on MS patients (van Langelaar et al., 2020). On the contrary, abnormal secretion of pro-inflammatory cytokines by T cells can lead to CNS inflammation and MS also worsens accordingly (Kaufmann et al., 2021). Experimental autoimmune encephalomyelitis (EAE) is a commonly used model to study MS (Hoffman et al., 2023). Study on GF mice has found that an increase in T cells and a decrease in Th1 and Th17 cell populations can alleviate EAE (Ochs et al., 2022). In addition, study has shown that the gut microbiota can alleviate the progression of MS by regulating innate immune signals in CNS (Rutsch et al., 2020). Through the detection of gut microbiota, it was found that the microbial community of MS patients showed a greater pro-inflammatory trend, and the depletion of certain microbial components may further increase the risk of MS recurrence (Tremlett and Waubant, 2018). Furthermore, the gut microbiota of MS patients can enhance the T cell response to inflammation in GF mice, further leading to the deterioration of EAE (Berer et al., 2017). Study has found that certain probiotics can increase the abundance of gut microbiota and alleviate the severity of MS (Tankou et al., 2018). In summary, the immune system and gut microbiota are closely related to MS. these findings provide a foundation for future research on the treatment of MS.

### 4.4 Autism spectrum disord

Autism spectrum disorder (ASD) is a verbal communication and behavior disorder caused by neurodevelopmental disorders. Its pathogenesis involves genetics, immune system, environment, intrauterine environment and other factors (Lord et al., 2018). ASD is highly correlated with inflammation. Research has found that the increase in LPS levels in the blood of patients with autism further promotes harmful substances to enter the brain and cause inflammation, further leading to the occurrence of ASD (Jang et al., 2022). The intestinal biopsy of autistic children showed that there were signs of infiltration of monocyte, lymphocytes, eosino­ phils and natural killer cell, and the level of proinflammatory cytokines in Astrocyte increased, indicating that inflammation was involved (Vuong and Hsiao, 2017). According to study, gut microbiota can regulate the pathological process of ASD, with reduced microbial diversity and increased biomass in the gut of ASD patients. The experiment found that the excessive growth of *Macromonas* and *Candida*, as well as the reduction of the proportion of *Bacteroidota* and the increase of the number of *Bacillota* promoted the occurrence of ASD (Zou et al., 2020). Recent studies have found that the abundance of *Bacteroidota*, *Bacillota*, *Pseudomonadota* and *Actinomycetota* had no significant correlation with the diagnosis of ASD, only *Streptococcus* and *Bifidobacteria* were proved to be related to ASD (Andreo-Martinez et al., 2022). Targeted regulation of gut microbiota may be a breakthrough in the treatment of ASD, but due to the inconsistency of the above results, it is difficult to accurately explain the results of the above studies. Further research is needed on the relationship between microbiota and ASD in the future.

### 4.5 Depression

Depression is characterized by emotional impairment, cognitive impairment, and even suicide, which are common in worldwide (Peirce and Alvina, 2019). Abnormal brain structure and function in hippocampus and prefrontal cortex are the inducements of depression (Idunkova et al., 2023). In addition, neurotransmitter disorder, endocrine disorder, reduction of neurotrophin, excessive pro-inflammatory cytokines and other factors can lead to depression (Song and Kim, 2021). With the deepening of study, more and more attention has been paid to the mechanism of chronic inflammation affecting the pathophysiology of depression (Woelfer et al., 2019). The high levels of inflammatory cytokines such as TGF-β, TNF-α, and IL-1β in the peripheral blood of patients with depression fully indicate that inflammation is related to depression (Bhatt et al., 2023). Moreover, the gut microbiota plays a potential regulatory role in depression. The study found that fluoxetine directly and indirectly changed the gut microbiota of patients while treating depression (Sun et al., 2019). Further study found that the greater richness and α diversity of gut microbiota is associated with depression (Madan et al., 2020). The level of *Enterobacteriaceae* and *Alisma* in stool samples of patients with depression increased, while the level of *Faecalibacterium* decreases (Jang et al., 2022). It can be inferred that changes in the gut microbiota may promote harmful bacterial infections, induce systemic inflammation, and ultimately lead to depression.

### 4.6 Psoriasis

Immune-mediated disorders can lead to psoriasis, characterized by abnormal itching and the appearance of squamous plaques on the skin (Nadeem et al., 2017). When psoriasis occurs on the hands or feet, treatment is more difficult (Merola et al., 2018). Increasing studies have shown that there are abnormal concentrations of inflammatory cytokines in psoriasis. Psoriasis can increase the levels of inflammatory cytokines in the blood and cerebrospinal fluid (Aleem and Tohid, 2018; Conti et al., 2021). It is found from the study of MGB axis that emotional is closely related to skin inflammation (Chen G. et al., 2021). With the deepening of study, it has been found that psoriasis may lead to depression, which in turn further increases the levels of pro-inflammatory cytokines and exacerbates the condition of psoriasis (Gonzalez-Parra and Dauden, 2019). Studies have found that TNF-α blockers used in the treatment of psoriasis patients may help alleviate their depressive symptoms, further indicating the potential connection between psoriasis and depression (Lian et al., 2020). Further research is needed on the mechanism of action between psoriasis and depression in the future.

### 4.7 Irritable bowel syndrome

The main characteristics of IBS are abdominal pain, bloating, and behavior, and it is the most common functional bowel disease worldwide (Maaser et al., 2019). IBS patients are divided into four subtypes: predominant constipation (IBS-C), predominant diarrhea (IBS-D), IBS with mixed bowel habits (IBS-M) and unclassified IBS (Su et al., 2023). IBS patients usually suffer from nervous system disease, but its pathological mechanism is still unclear (Ancona et al., 2021). It has been reported that it may be related to neuroendocrine response, abnormal intestinal secretion, gut microbiota alterations, intestinal permeability, disordered gut motility, immunomodulation, and other factors (Jeffery et al., 2020). Recent studies have emphasized that the MGB axis can have a positive effect on the treatment of IBS by improving immune function (Hillestad et al., 2022). IBS can promote the infiltration of immune cells, and T and B cells are released throughout the body with the circulatory system, leading to inflammatory reactions in the body (Ng et al., 2018). The intestinal mucosal immune system needs to use mast cell as the communication medium to establish contact with the nervous system. In this process, mast cell promote endocrine cells and neurogen to release neurotransmitters by secreting inflammatory mediators, causing CNS reaction, which in turn leads to high sensitivity of the ENS and induces IBS (Ozcaglayan et al., 2020). In addition, changes in gut microbial diversity and abundance are involved in the pathogenesis of IBS, with an increase in the abundance of *Bifidobacteria* and *Lactobacilli* in IBS patients (Altomare et al., 2021). When the feces of IBS-D patients are transferred to GF mice, it can change the digestive function and intestinal barrier, and the innate immune system is activated to cause anxiety (De Palma et al., 2017). This indicates the importance of the MGB axis in the alleviate of IBS, and regulating the immune system and gut microbiota may be effective in treating IBS.

### 4.8 Inflammatory bowel diseases

More than 6.8 million people worldwide suffer from IBD, including 2.5 million to 3 million people in Europe. The number of people suffering from IBD in emerging countries in South America, Asia and Africa also continues to increase (Adolph et al., 2022). IBD includes Krohn’s disease (CD) and ulcerative colitis (UC), up to 70% of IBD patients experience abdominal pain symptoms, while approximately 20-60% of IBD patients experience persistent pain (Sinopoulou et al., 2021). IBD can cause damage to the intestinal mucosal barrier and an increase in bacterial infiltration and mucolytic bacteria, and also alter the gut microbiota (Kim et al., 2019). The main characteristics of gut microbial ecological imbalance in IBD patients are the decrease of *Firmicutes* and *Bacteroidetes* abundance and the relative increase of *Proteobacteria* species (Zuo and Ng, 2018). However, the relationship between IBD and gut microbiota disorders is complex. According to the studies, microecological disorders may precede the occurrence of IBD and are an important factor triggering IBD (Pittayanon et al., 2020). However, some studies hold opposing views, believing that microecological disorders may not be a triggering factor for IBD, but may evolve during the continuous stages of the disease (Jin et al., 2021). Therefore, further study is needed on the interrelationship between microecological disorders and IBD. It is reported that gut microbiota can increase the incidence rate of IBD by regulating the development of ENS, and the interaction between MGB axis may be related to the severity of IBD symptoms (Nguyen et al., 2020). It is speculated that IBD patients are accompanied by mental illness, which may be caused by changes the function of the gut microbiota, thereby affecting the stability of the MGB axis (Labanski et al., 2020). In addition, the saprophytic microbiota may potentially affect the expression of IBD by affecting the vagal inflammatory reflex (Bonaz et al., 2017). Other studies have found a correlation between the degree of inflammation and SCFAs uptake and bacterial metabolism profiles in IBD patients (Lavelle and Sokol, 2020). It is speculated that IBD may affect the nervous system through the MGB axis, thereby inducing neuroinflammation. Furthermore, exploring the relationship between IBD and gut microbiota may be a future study direction.

## 5 Innate immune signaling pathway regulates inflammatory and infective diseases

With the deepening of research, it is urgent to explore the relationship between innate immune signaling pathways and inflammation. Infection with eliminate xenobiotics and disruption of immune system homeostasis can lead to inflammation in the body (Netea et al., 2017). The gastrointestinal tract is the intersection of bidirectional communication between the immune system, nervous system, and microbiota. Studies have found that gut microbes can regulate the innate immune system, and then affect the structure and function of nerves system. The imbalance of the immune system can induce the occurrence of inflammatory and infective diseases, in which the innate immune signaling pathway plays a crucial regulatory role in this process (Liu et al., 2020a). It is speculated that the innate immune signaling pathway may be involved in the regulation of the MGB axis, thereby affecting inflammatory and infective diseases.

### 5.1 Inflammasome signaling pathway

Inflammasome is an innate immune complexes, and their activation can suppress inflammation caused by danger signals generated by gut microbiota. Currently, research has found that PRRs from different families play an important role in the activation of inflammasomes. Furthermore, the activation of inflammasome leads to the release of IL-18 and IL-1β, which helps prevent the occurrence of intestinal inflammation (Mukherjee et al., 2020). The combination of TLR2 and α-synaptic promotes the activation of neurotoxic signals, leading to the production of inflammatory factors, and ultimately triggers inflammatory response (Zheng M. et al., 2021). NLRC5 can control the homeostasis of innate immune system and inhibit inflammation by reducing NF-κB signaling pathway (Nyul-Toth et al., 2017). Defects in NLRP6 inflammasome signaling can lead to abnormal colonization of gut microbiota and may lead to inflammatory diseases driven by ecological imbalances (Bao et al., 2019). GPR43 can reduce intestinal inflammation by activating NLRP3 inflammasome (Fujiwara et al., 2018). Activated inflammasomes and IL-1β, IL-6, and IL-18 play important roles in regulating major depressive disorders (Chan et al., 2019). It is worth noting that NLRP3 inflammasomes are crucial in regulating and shaping peripheral and CNS inflammation diseases (Pellegrini et al., 2020). Other studies have showed that various stimuli caused by neuroinflammatory or degenerative processes can promote the activation of NLRP3 inflammasome, thereby altering the gut microbiota (Pellegrini et al., 2017). It was found that the knockout of NLRP3 could lead to the increase of *Firmicutes* and a decrease in *Bacteroidetes* in the gut of mice, as well as the increase of *Lachnospiraceae*, *Ruminococcaceae* and *Prevotellaceae* (Gao et al., 2023). Collectively, inflammasome may be the key target for directly regulating inflammation and infectious diseases. Exploring the relationship between inflammasome and diseases may be the key to effectively alleviate inflammatory and infective diseases.

### 5.2 IFN-I signaling pathway

IFN-I participates in various immune regulatory functions in the host and can resist inflammatory diseases caused by external microbial invasion. IFN-I is a major antiviral molecule in the innate immune system (Crow, 2023). When PRRs recognizes foreign products, pathogens or molecules related to injury, IFN-I and other cytokines necessary for effective host defense are activated, and then participate in the inhibition of inflammation (Kato et al., 2017). However, when IFN-I production increases abnormally, immune disorders and inflammatory diseases also occur (Qiu et al., 2023). IFN-I can effectively treat autoimmune diseases by inhibiting inflammasome signaling and regulating inflammation by recruiting T cells (Sumida et al., 2022). At present, studies on the regulation of microbiota by IFN-I have been widely reported. *Lactobacillus acidophilus* induce the antiviral response of IFN-I through TLR-2 dependency in murine bone marrow (Si et al., 2022). The metabolites produced by *Clostridium orbiscindens* can alleviate the invasion of influenza in mice by enhancing IFN-I pathway (Steed et al., 2017). Protective microbiota-dependent IFN-I signaling is blocked by autophagy proteins (Martin et al., 2018). IFN can also regulate gut microbiota to varying degrees, indicating the importance of bidirectional interactions between microbiota and IFN-I pathway in regulating immune responses to pathogen attacks, and this potential interaction may also affect the function of CNS (Giles and Stagg, 2017). It is reported that IFN-I signaling promotes the severity of AD by inhibiting the occurrence and brain function of hippocampal neurogenesis, while reducing neuroinflammation associated with IFN-I may alleviate the progression of this disease (Taylor et al., 2018). The increase of IFN-I promotes neuroinflammatory response and disease progression in PD mouse models, and similar findings are also found in the postmortem brain of PD patients. When IFN-I signal transduction is eliminated, neuroinflammatory reaction can be reduced, which provides direct evidence for IFN-I signal transduction to participate in PD (Chen K. et al., 2022). Similarly, the absence of IFN-I signaling transcription factors can lead to the infiltration of inflammatory factors into the CNS, leading to an exacerbation of EAE in mice (Kronborg et al., 2022). It is speculated that IFN-I may affect inflammatory and infective diseases by participating in the physiological process of MGB axis.

### 5.3 NF-κB signaling pathway

NF-κB family is detected in almost all tissues, which can maintain the stability of innate immune system and adaptive immunity (Guldenpfennig et al., 2023). NF-κB is the “main switch” that promotes the expression of inflammation related genes, playing an important role in regulating and activating inflammatory intermediates when inflammation occurs (Singh et al., 2020). When NF-κB is activated, it can aggravate inflammation by triggering inflammatory molecules such as TNF, LPS and IL-1, and activating T cells, B cells and other cell surface receptor such as TLRs, TNF receptors and IL-1 receptors (Hu et al., 2020). The imbalance of gut microbiota composition can cause various inflammatory diseases through the NF-κB signaling pathway (Liu B. et al., 2020). For example, When the gut microbiota is dysregulated, it increases the risk of *Campylobacter jejuni* invasion, thereby activating the NF-κB pathway to stimulate the immune system (Tang et al., 2021). Conversely, strains of *Lachospiraceae* can protect against inflammatory diseases induced by high-fat diet induced activation of inflammasome by inhibiting the NF-κB/MAPK signaling pathway (Truax et al., 2018). Neuroinflammatory diseases such as AD and PD are all related to NF-κB signaling pathways (Xu et al., 2023). NF-κB activation can induce neuroinflammatory diseases, which is achieved by inducing microglia to produce proinflammatory factors (Cai et al., 2022). In addition, the disorder of gut microbiota can lead to the activation of NF-κB in the hippocampus, which aggravates neuroinflammation and anxiety like behavior in animal models (Xu et al., 2021). Similarly, colitis can cause the increase of NF-κB in the gut and hippocampus, and lead to severe memory impairment. These symptoms will be alleviated when the disordered gut microbiota recovers (Jang et al., 2018). The NF-κB signaling pathway is unquestionable in regulating inflammatory diseases, but its relationship with the MGB axis needs further exploration.

### 5.4 PARK7/DJ-1 signaling pathway

PARK7/DJ-1 is a peroxisome, which is expressed in almost all types of cytoplasm and has the function of protecting cells from oxidative stress (Zhang L. et al., 2020). It is reported that PD can change the expression of PARK7/DJ-1 in the brain. With the deepening of study, it has been found that PARK7/DJ-1 can regulate almost all neuroinflammatory diseases. According to the experimental data, PARK7/DJ-1 alleviates the condition of AD by reducing the activity of glyoxalase activity and reducing the harmful effects on neurons (Pap et al., 2022). It is reported that the decrease in the expression of PARK7/DJ-1 induces an inflammatory response in colon cancer cells, indicating its anti-inflammatory effect (Lippai et al., 2021). Similarly, PARK7/DJ-1 affects the local and systemic inflammatory characteristics of IBD by maintaining gut microbiome and mucosal integrity (Pap et al., 2022). In addition, through the proteomics detection of the plasma of IBD patients, it was found that the PARK7/DJ-1 protein in plasma increased (Di Narzo et al., 2019). On the contrary, Zhang et al. found that compared to healthy subjects, the levels of PARK7/DJ-1 in the intestine of CD or UC patients decreased (Zhang J. et al., 2020). Due to differences in disease, samples, and degree of inflammation, there are obvious contradictions in the above reports, therefore more research is needed.

## 6 Therapeutically targeting the microbiota gut brain axis

**Figure 4:**
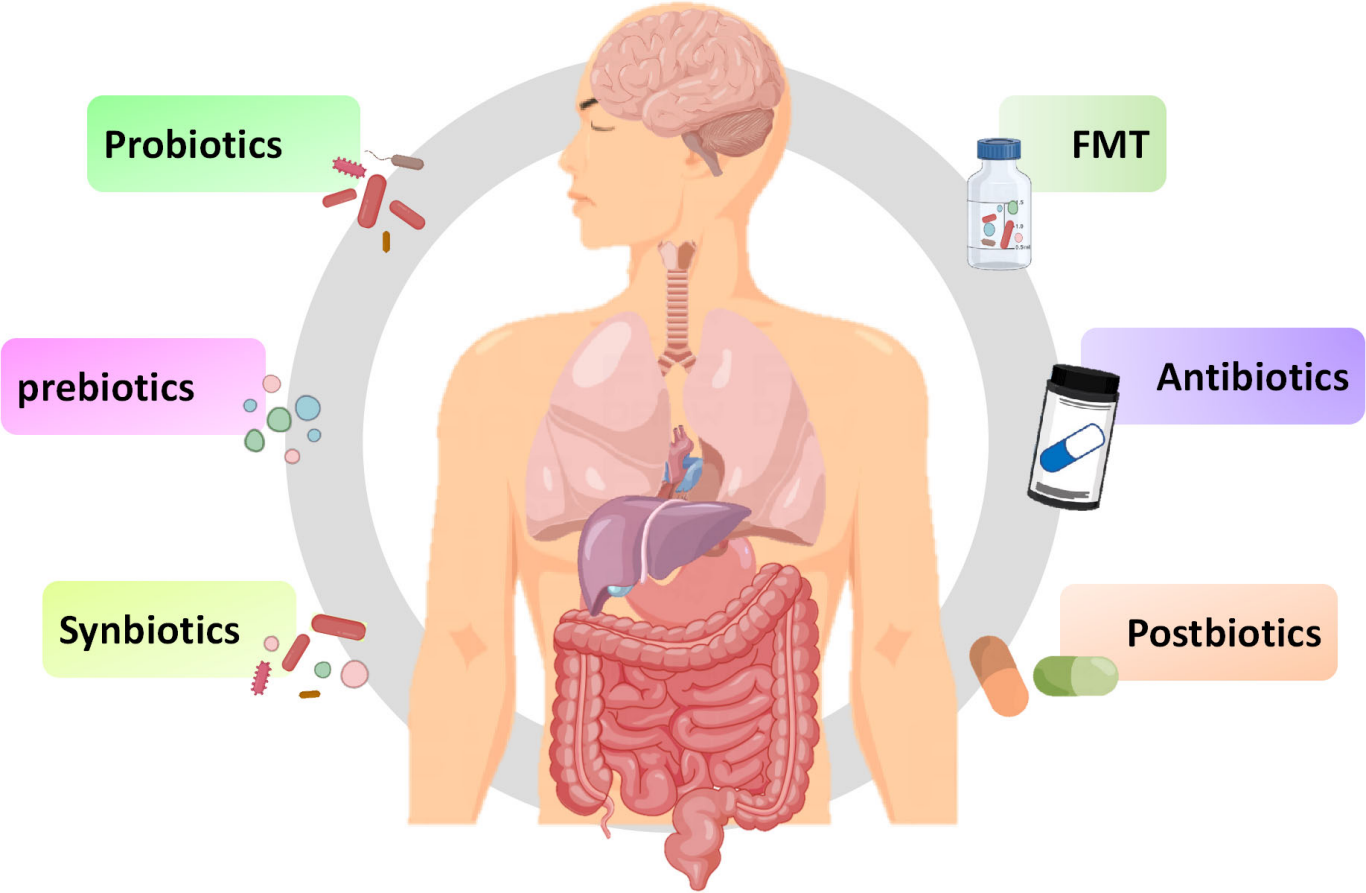
Potential therapies for inflammation based on gut microbiota. Fecal microbiota transplantation, antibiotics, probiotics, prebiotics, synbiotics and postbiotics are potential treatment methods for inflammation based on the MGB axis.

In addition to exploring the relationship between innate immune signaling pathways and the MGB axis, exploring methods using the MBG axis as a therapeutic target is also increasingly being extensively studied. As a key link in the MGB axis, intestinal microbiota may be a breakthrough in regulating inflammatory and infective diseases. The methods of regulating gut microbiota, including probiotics, prebiotics, synbiotics, and prebiotics, have also been widely studied for their effects on inflammatory and infective diseases (**Figure 4**). Although there have been numerous reports on the positive effects of regulating gut microbiota, various methods also have some limitations, making their effectiveness still open to discussion.

### 6.1 Fecal microbiota transplantation

Fecal microbiota transplantation (FMT) involves transferring a small amount of liquefied or filtered feces to the subject. FMT, as a potential treatment method, has a positive effect on treating inflammation (Johnsen et al., 2018). Sun et al. found that FMT can reduce the function of microglia and astrocyte by reducing TLR4/TNF-α signaling pathway (Sun et al., 2018). Study has found that FMT can temporarily improve leg tremors and neuroinflammation caused by PD (Huang et al., 2019). Furthermore, transplanting fecal microbiota from PD patients to mice over expressing α-synuclein will aggravate motor function problems, indicates that gut microbiota is related to PD (Sampson et al., 2016). FMT can improve the symptoms of children with ASD and improve intestinal manifestations such as constipation, diarrhea, and indigestion, possibly by increasing the diversity of gut microbiota (Kang et al., 2017). Numerous clinical studies have shown that the decrease in abundance and diversity of gut microbiota promotes the occurrence of depression (Liu et al., 2020b). FMT can improve depression by regulating the diversity of gut microbiota (Kurokawa et al., 2018). In studies related to depression, it has been found that FMT in healthy animals reduces depressive symptoms in animals with spinal cord injury, while FMT in individuals with depression can induce depressive behavior in GF mice (Pearson-Leary et al., 2020; Schmidt et al., 2020). Based on the above research, FMT may target the gut brain axis to alleviate depressive symptoms. During the FMT process, there may be potential pathogenic bacteria or viruses that may disrupt beneficial microbiota, and it is still clouded with uncertainties (Metta et al., 2022). Therefore, before large-scale clinical application, a lot of exploration is needed to determine the effectiveness and safety of therapeutic procedures.

### 6.2 Antibiotics

Antibiotics could enhance immune response by affecting gut microbiota. It is reported that antibiotics such as amoxicillin azithromycin, clarithromycin or ciprofloxacin may alleviate the condition of ASD by affecting the gut microbiota (Braakman and van Ingen, 2018). In addition, antibiotics may have positive or negative effects on the treatment of IBD and IBS (Lee et al., 2020). Although antibiotics can alter the gut microbiota, unlike FMT, antibiotics may have a negative impact on the gut microbiota. It is reported that the administration of antibiotics can kill most of the resident microbiota in the intestine, providing space for the development of pathogens, leading to ecological imbalance of the microbiota (Duan et al., 2022). The use of antibiotics can cause disruption of the gut microbiota, leading to nervous system disease. It is confirmed that the used of penicillin and quinolones can increase the risk of depression and anxiety (Aygun, 2020). The direction of changes in gut microbiota depends on the type of antibiotic used. For instance, macrolides can reduce the abundance of *Actinobacteria*, oral vancomycin can reduce *Firmicutes* and increase *Proteobacteria*, while penicillin does not cause significant changes to the gut microbiota (Fan et al., 2022). Considering the harmful effects of antibiotics on the homeostasis of gut microbiota, special attention should be paid to their dosage when using them.

### 6.3 Probiotics

*Bifidobacterium* and *Lactobacillus* are common probiotics, which are living microorganisms, and yogurt in daily food contains a large amount of probiotics (Yan and Polk, 2020). Multiple studies have shown that probiotics containing *Bifidobacterium* and *Lactobacillus* can improve the cognitive impairment of AD patients and reduce the motor dysfunction of PD mouse models (Naomi et al., 2021). Sun et al. found that *Clostridium butyricum* may have neuroprotective effect on PD mice through the gut brain axis (Sun et al., 2021). Similarly, *Lactobacillus acidophilus, Bifidobacterium bifidum, Lactobacillus reuteri*, and *Lactobacillus fermentum* can improve motor dysfunction in patients with PD (Tamtaji et al., 2019). In study on depression, it was found that depression can reduce the abundance of *Bifidobacterium* and *Lactobacillus*, while administering *Lactobacillus* and *Bifidobacterium* isolated from healthy feces could alleviate the symptoms of depression in mice (Jang et al., 2019). Interestingly, *Eubacterium, Ruminococcaceae, Erysipelothrix and Spirillaceae* may be beneficial to autistic patients (Liu et al., 2019). Three months of administration of *Lactobacillus acidophilus, Lactobacillus rhamnosus*, and *Bifidobacterium longum* can alleviate the severity of autism (Shaaban et al., 2018). Overall, utilizing probiotics as a method of inflammatory diseases has great potential. However, there are significant differences in the composition, stability, and authenticity of probiotics, and there is no consensus on dosage, duration, and type of probiotics consumed. Additionally, probiotics typically do not reside in the intestine and require daily intake to achieve their stable effects.

### 6.4 Prebiotics

Prebiotics are defined as a “substrate selectively utilized by host microorganisms conferring a health benefit” (Swanson et al., 2020). Prebiotics are composed of inulin, fructo-oligosaccharides, galacto-oligosaccharides, and resistant starch. Daily foods including fruits, vegetables, grains, and milk contain a large amount of prebiotics (Bamigbade et al., 2022). Although prebiotics cannot be absorbed by intestine, they can be utilized by certain gut microbiota, directing the microbiota towards the beneficial direction of the host (Verkhnyatskaya et al., 2019). Probiotics can affect the activity of the CNS and regulate inflammatory responses in a similar manner to probiotics by affecting the composition of the gut microbiota (Carlessi et al., 2021). In an open study, it was found that the combination of papain and pepsin could alleviate the symptoms of ASD (Song et al., 2022). Dong et al. reported that polymannuronic acid improved the motor function of mice with PD symptoms, inhibits intestinal, brain and systemic inflammation, and improves the integrity of intestinal tract and BBB (Dong et al., 2020). From this, it can be seen that prebiotics can alleviate inflammation to a certain extent, but their beneficial effects still require extensive research.

### 6.5 Synbiotics

Synbiotics are a combination of prebiotics and probiotics, defined as “amixture comprising live microorganisms and substrate(s) selectively utilized by host microorganisms that confers a health benefit on the host” (Swanson et al., 2020). Synbiotics can enhance the effectiveness of prebiotics and probiotics, enabling them to have synergistic effects and maximize their beneficial effects on host health (Trone et al., 2023). Synbiotics can enhance intestinal function by regulating microbiota (Li et al., 2021). In addition, synbiotics can reduce the risk of postoperative infection in patients by improving immune system function (Yadav et al., 2022). Taking synbiotics containing *Bifidobacterium infantis, Lactobacillus rhamnosus, Bifidobacterium lactis, Lactobacillus paracasei* combined with fructooligosaccharides has a positive effect on ASD patients by increasing the levels of beneficial bacteria and reducing the levels of pathogenic bacteria (Wang Y. et al., 2020). In addition, synbiotics containing GOSs, L. helveticus and B. longum have a positive impact on the process of depression (Kazemi et al., 2019). However, there are still many issues to be addressed regarding the beneficial effects and application prospects of synbiotics. For example, the optimal combination method, optimal ratio, and dosage of probiotics and probiotics require in-depth research.

### 6.6 Postbiotics

Postbiotic is bioactives produced by bacteria themselves or through interactions with the host, among which SCFAs and intestinal peptide are common postbiotic. At present, the research of postbiotic in treating inflammatory diseases is limited. However, recent reports have described the general effects of microbiome and SCFAs on neurological diseases through the gut brain axis (van de Wouw et al., 2018). Studies have found that polyunsaturated fatty acid supplementation can alleviate the main symptoms of autism (Cheng et al., 2017). Uridine, DHA and choline intake can alleviate colitis by reducing the accumulation of α-synaptic (Perez-Pardo et al., 2018). The use of propionic acid therapy can induce autism like phenotypes in rats (Sharma et al., 2022). Intestinal peptides have a recognized role in affecting neurological diseases. The role of gut peptides in regulating neurological disease is generally recognized, but they may be resisted during use as MGB axis signals (Lach et al., 2018). As a postbiotic, the special structural components of heat-killed probiotics is benefit for host, and many studies have proved that they can regulate the MGB axis (Wu et al., 2022). Heat-killed probiotics can extend shelf life and potentially improve safety, thus having a significant market advantage compared to probiotics. Furthermore, fully utilizing the beneficial bioactive substances of the gut microbiota may be an effective method to replace probiotics.

## 7 Conclusion

With a deeper understanding of the MGB axis, new avenues have been opened for understanding the treatment of inflammatory and infective diseases. To understand the regulatory role of MGB axis in diseases, it is first necessary to understand the structure of MGB and its communication mechanism. The nervous system, immune barrier, gut microbiota, and metabolites in the MGB axis are crucial for regulating inflammatory and infective diseases. As an important part of the MGB axis, the gut microbiota may be an important breakthrough point. The concept of MGB axis involvement in inflammatory diseases is increasingly recognized. Numerous clinical and study results showed alterations in the gut microbiota composition in PD, AD, MS, IBS, and IBD. Perhaps due to individual differences, gut microbiota of the same disease may exhibit differences, the causal relationship between the two is highly uncertain. Inconsistent results may also be attributed to treatment status, diet, and age. In addition, techniques such as sample collection and storage, DNA extraction methods, primer selection, sequencing methods, and bioinformatics analysis can also affect the results. At the same time, the small sample size in most studies is also a factor that cannot be ignored. Therefore, attention should be paid to the above influencing factors when collecting, testing, and analyzing samples. In addition, exploring the innate immune signaling pathway related to the MGB axis and diseases is crucial for gaining a deeper understanding of its pathogenesis, and is also an effective method for accurately treating inflammatory and infective diseases. Moreover, due to the diverse potential mechanisms targeting MGB axis, caution must be exercised in the treatment of inflammatory and infective diseases to reduce risks. In addition, regulating the MGB axis to treat diseases has become a current research hotspot, and the therapeutic effects of FMT, antibiotics, probiotics, and prebiotics have also been verified. However, their limitations also affect their promotion and application in the market. At present, there is an increasing number of over-the counter probiotic and prebiotic diets in the market, raising public awareness of how they regulate health has become particularly important.

## Author contributions

CY: Data curation, Investigation, Methodology, Writing – original draft. YH: Conceptualization, Formal Analysis, Methodology, Writing – original draft. KX: Formal Analysis, Methodology, Software, Writing – review & editing. LF: Data curation, Methodology, Software, Writing – review & editing. SG: Project administration, Resources, Supervision, Writing – review & editing. LC: Funding acquisition, Project administration, Supervision, Writing – original draft.
